# Potential Hepatic Lipid Markers Associated with Nonalcoholic Steatohepatitis and Fibrosis in Morbid Obesity Patients

**DOI:** 10.3390/jcm12113730

**Published:** 2023-05-29

**Authors:** Hua-Chien Wu, Yin-Ru Hsieh, Weu Wang, Ching-Wen Chang, I-Wei Chang, Chi-Long Chen, Chun-Chao Chang, Chia-Hsuan Chang, Wei-Yu Kao, Shih-Yi Huang

**Affiliations:** 1 Division of Gastroenterology and Hepatology, Department of Internal Medicine, School of Medicine, College of Medicine, Taipei Medical University, Taipei 110, Taiwan; b101106061@tmu.edu.tw (H.-C.W.); chunchao@tmu.edu.tw (C.-C.C.); 2 School of Nutrition and Health Sciences, Taipei Medical University, Taipei 110, Taiwan22491@s.tmu.edu.tw (C.-H.C.); 3 Division of Digestive Surgery, Department of Surgery, Taipei Medical University Hospital, Taipei 110, Taiwan; wangweu@tmu.edu.tw; 4 Department of Surgery, School of Medicine, College of Medicine, Taipei Medical University, Taipei 110, Taiwan; 5 TMU Research Center for Digestive Medicine, Taipei Medical University, Taipei 110, Taiwan; 6 Graduate Institute of Metabolism and Obesity Sciences, Taipei Medical University, Taipei 110, Taiwan; changc11@tmu.edu.tw; 7 Laboratory of Human Carcinogenesis, Center for Cancer Research, National Cancer Institute, Bethesda, MD 247202, USA; 8 Department of Pathology, Taipei Medical University Hospital, Taipei 110, Taiwan; 9 Department of Pathology, School of Medicine, College of Medicine, Taipei Medical University, Taipei 110, Taiwan; 10 Department of Clinical Pathology, Wan Fang Hospital, Taipei Medical University, Taipei 110, Taiwan; 11 Department of Pathology, Shuang Ho Hospital, Taipei Medical University, Taipei 110, Taiwan; 12 Division of Gastroenterology and Hepatology, Department of Internal Medicine, Taipei Medical University Hospital, Taipei 110, Taiwan; 13 Taipei Cancer Center, Taipei Medical University, Taipei 110, Taiwan; 14 Nutrition Research Center, Taipei Medical University Hospital, Taipei 110, Taiwan

**Keywords:** nonalcoholic steatohepatitis, lipid profile analysis, liver fibrosis

## Abstract

This study investigated differences in lipidomic profile features in nonalcoholic steatohepatitis (NASH) between mild and significant liver fibrosis cases among patients with morbid obesity. Wedge liver biopsy was performed during sleeve gastrectomy and significant liver fibrosis was defined as a fibrosis score ≥ 2. We selected patients with NASH with non/mild fibrosis (stage F0–F1; *n* = 30) and NASH with significant fibrosis (stage F2–F4; *n* = 30). The results of the liver tissue lipidomic analysis revealed that the fold changes of triglyceride (TG) (52:6); cholesterol ester (CE) (20:1); phosphatidylcholine (PC) (38:0) and (50:8); phosphatidic acid (PA) (40:4); phosphatidylinositol (PI) (49:4); phosphatidylglycerol (PG) (40:2); and sphingomyelin (SM) (35:0) and (37:0) were significantly lower in patients with NASH with F2–F4 than those with NASH with F0–F1 (*p* < 0.05). However, the fold changes of PC (42:4) were relatively higher in patients with NASH with stage 2–4 fibrosis (*p* < 0.05). Moreover, predictive models incorporating serum markers levels, ultrasonographic studies, and levels of specific lipid components [PC (42:4) and PG (40:2)] yielded the highest area under receiver operating curve (0.941), suggesting a potential correlation between NASH fibrosis stages and liver lipid accumulation among specific lipid species subclasses. This study demonstrated that the concentrations of particular lipid species in the liver correlate with NASH fibrosis stages and may indicate hepatic steatosis regression or progression in patients with morbid obesity.

## 1. Introduction

Nonalcoholic fatty liver disease (NAFLD) is the most prevalent chronic liver disease worldwide and affects 20–30% of the general population [1,2]. NAFLD is a spectrum of chronic liver diseases, ranging from simple triglyceride (TG) accumulation, nonalcoholic steatohepatitis (NASH) that may progress to fibrosis, cirrhosis, and hepatocellular carcinoma (HCC) [3,4,5]. NASH, a progressive form of NAFLD, is characterized with pericellular fibrosis, which may evolve to cirrhosis or HCC if poorly controlled. All NAFLD stages are associated with significantly increased overall mortality, especially NASH with advanced fibrosis [6,7,8]. NAFLD and NASH, the hepatic manifestation of metabolic syndrome, is associated with central obesity, insulin resistance, hypertriglyceridemia, hypertension and cardiovascular disease as well [9,10]. The prevalence of NAFLD has been reported to be as high as 74–90% among patients with morbid obesity with a body mass index (BMI) higher than 35 kg/m^2^ [11]. The prevalence of NASH among patients with morbid obesity undergoing bariatric surgery ranges up to 98% in previous studies [12] and is 50.8–71.3% in Taiwan [13,14,15,16].

Lipotoxicity, defined as an abnormal cellular lipid composition leading to toxic lipid accumulation, organelle dysfunction, cell injury, and chronic inflammation, is characteristic of NASH [17]. However, triglycerides are primarily associated with these pathologies and other lipid moieties seem to be involved in the development and severity of NAFLD. An unbalanced ratio between ceramides and terminal metabolic products in the liver and plasma promotes weight gain, inflammation, and insulin resistance. Some sphingolipid species, such as ceramides (long-chain dihydroceramide C22:0), may be biomarkers for NAFLD [18]. Caussy et al. reported that a combination of 10 serum metabolites (including taurine, fucose, palmitoleate, etc.) can be used to diagnose advanced fibrosis with greater accuracy than fibrosis-4 (FIB-4) score or NAFLD fibrosis score (NAFLD-FS), and may be a useful tool to screen at-risk patients for advanced disease [19]. Previous studies have demonstrated liver and serum lipidomic change, including significant increase in triglyceride, diacylglycerols, and sphingolipids, in patients with NAFLD and NASH [20,21,22]. However, there is a need for additional study characterizing the correlation of lipid metabolites with NASH and fibrosis stage and identifying the progression of NASH, to more effectively prevent exacerbation to HCC [23].

This prospective cohort study used lipidomic analysis to investigate lipidomic profile features of NASH with non/mild and significant liver fibrosis in patients with morbid obesity.

## 2. Materials and Methods

### 2.1. Study Design and Protocol

This prospective study involved 200 patients with morbid obesity who received laparoscopic sleeve gastrectomy at Taipei Medical University Hospital between October 2016 and December 2020. This study, approved by the Joint Institutional Review Board of Taipei Medical University (TMU–JIRB No.: N201203002 and N201601029), was conducted in accordance with the Declaration of Helsinki. Written informed consent was obtained from all participants.

The inclusion criteria were: age of 20–65 years and BMI over 37.5 kg/m^2^, over 32.5 kg/m^2^ with a comorbidity other than diabetes, or over 27.5 kg/m^2^ with poorly controlled diabetes [24]. The exclusion criteria were: end-stage organ damage; pregnancy; previous bariatric surgery; prolonged exposure to known hepatotoxins, such as alcohol and drugs; and other causes of chronic liver disease, including hepatitis B virus, hepatitis C virus, hepatitis D virus, human immunodeficiency virus infection, autoimmune hepatitis, primary biliary cirrhosis, primary sclerosing cholangitis, Wilson disease, and hemochromatosis.

During laparoscopic sleeve gastrectomy, all patients received a wedge liver biopsy using laparoscopic guidance. Liver tissue specimens were fixed in 10% formalin, embedded in paraffin, and then stained with haematoxylin and eosin for histopathological analysis. Two experienced pathologists, who were unaware of the patients’ identity and history, read histological slides and coded them; all codes were finalised based on a consensus between the two pathologists. A steatosis, activity, and fibrosis score were given for each patient for the diagnosis of NASH, as was done in Bedossa’s study [25]. Advanced liver fibrosis was defined by a fibrosis score of 3. Written informed consent was obtained from all patients who received surgery. This study was approved by the Taipei Medical University–Joint Institutional Review Board (TMU–JIRB No.: N201601029) (clinical trial number: ClinicalTrials.gov identifier NCT04059029) [16].

From the 200 patients with morbid obesity, 60 samples with NASH with stage 0–1 fibrosis (*n* = 30) and NASH with stage 2–4 fibrosis (*n* = 30) were obtained for liver lipidomic analysis.

### 2.2. Noninvasive Serum Markers

Venous blood samples were collected after overnight fasting. Fatty liver index (FLI) was calculated using the following formula: FLI = (e 0.953 × loge (TG) + 0.139 × BMI + 0.718 × loge (gamma-glutamyltransferase [26]) + 0.053 × waist circumference [27] − 15.745)/(1 + e 0.953 × loge (TG) + 0.139 × BMI + 0.718 × loge (GGT) + 0.053 × WC − 15.745) × 100 [28]. The aspartate aminotransferase/platelet ratio index (APRI) was calculated as (aspartate aminotransferase (AST) [29] f/platelet counts [109/L]) × 100 [30]. NAFLD-FS was calculated using the following formula: −1.675 + (0.037 × age [years]) + (0.094 × BMI) + (1.13 × hyperglycemia or diabetes [yes = 1, no = 0]) + (0.99 × AST/alanine aminotransferase [ALT]) − (0.013 × platelet [109/L]) − (0.66 × albumin [g/dL]) [31]. FIB-4 score was calculated using the following formula: (age [years] × AST [U/L]/platelet [109/L] × √ALT [U/L]) [32].

### 2.3. Ultrasonographic and Transient Elastography Examination

Liver stiffness measurement (LSM), controlled attenuation parameter (CAP) measurements using transient elastography (FibroScan), ultrasonographic (US) fatty score, and US fibrosis score with abdominal sonography were completed, as in a previous study [16]. This provides a 90% of prediction accuracy to identify patients who are less likely to progress to advanced in liver biopsies.

### 2.4. Liver Sample Preparation

To prevent the decellularized liver scaffold proteins from interfering with the release of bioactive molecules and liver functionality, the liver samples were homogenized and processed using the Tarek Saleh et al. methodology, with partial modification [33]. The liver homogenate was separated using centrifugation (3000 rpm, 4 °C, 10 min) and maintained at −80 °C until analysis. Crude lipids were then extracted using a modified Folch method [34,35]: 3 mL of chloroform and 1.5 mL of methanol were added to a 200-mg liver sample in a 15-mL centrifuge tube; the contents were gently vortexed after each addition. Subsequently, 1.25 mL of distilled deionised water was added to the mixture, which was then incubated for 1 h at room temperature. The mixture was then centrifuged at 3000 rpm for 10 min at 4 °C. The organic phase was subsequently dried in a vacuum concentrator at the room temperature.

### 2.5. Ultrahigh-Performance Liquid Chromatography–Tandem Mass Spectrometer

The organic layer of the Folch extraction was reconstituted in an isopropanol (IPA)/acetonitrile (ACN)/H_2_O (2:1:1) solution and subjected to ultrahigh-performance liquid chromatography–tandem mass spectrometer (UPLC–MS/MS) analysis. We used an ACQUITY UPLC system (Waters Corporation, Milford, MA, USA) coupled with a SYNAPT G2 Q-ToF mass spectrometer (Waters Corporation, Milford, MA, USA). A quality assurance (QA) sample was prepared by pooling 5 µL from each vial. This QA sample was injected throughout the analytical batch for normalization and correction purposes. Samples were randomized and injected, together with QA extracts, onto a UPLC system.

UPLC separations were performed with an ACQUITY CSH C18 column with a particle size of 1.7 µm and measuring 2.1 × 100 mm (Waters Corporation, Milford, MA, USA). The column temperature was maintained at 55 °C and eluted at a flow rate of 0.4 mL/min, with an injection volume of 5 µL. Lipid species were separated chromatographically over mobile phases (A) 60% ACN–40% H_2_O containing 10 mM ammonium formate and 0.1% formic acid and (B) 90% IPA–10% ACN containing 10 mM ammonium formate and 0.1% formic acid. The gradient profile for positive ionisation detection was initially 40% B and was maintained for 2 min. It increased linearly to 50% B in 0.1 min, to 54% B in 9.9 min, to 70% B in 0.1 min, to 99% B in 5.9 min, and then decreased to the initial ratio in 0.1 min and stayed at this level for 1.9 min. The eluent was directly introduced to the MS/MS.

The mass spectrometer parameters indicating positive ionisation were as follows: desolvation gas, 900 L/h; desolvation temperature, 550 °C; cone gas, 15 L/h; source temperature, 120 °C; capillary voltage, 2.8 kV; cone voltage, 40 V; and time-of-flight mass spectrometry, scan range 100–2000 *m*/*z*. The data acquisition rate was set to 1.2 s by using the Waters MSE acquisition mode, with full exact data on mass collected simultaneously through the rapid alternation between two functions. Function 1 was used to acquire data with a low collision energy of 4 and 2 eV for the trap and transfer collision cells, respectively, and Function 2 was used to acquire data with a transfer collision energy ramp of 15–35 eV. Leucine–enkephalin was used as the lock mass at a concentration of 1 ng/μL and flow rate of 5 μL/min. Data were collected in continuum mode; the lock spray frequency was set at 20 s. All data acquisition was controlled using Waters MassLynx v4.1 software.

### 2.6. Untargeted Lipidomic Analysis

After obtaining the UPLC–MS/MS raw data of all samples, lipidomics data were first processed and later imported into Progenesis QI software for small molecules and lipids (Nonlinear Dynamics, Waters Corporation, Milford, MA, USA). Progenesis QI facilitated quantifying and identifying small lipid molecules based on the Human Metabolome Database. The lipidomics information, including retention time, isotope patterns, and error mass were provided. The raw abundance value of each identified lipid was normalised according to the database. Data on the analytical compounds, such as mass similarity, retention time similarity, and fragmentation score, were retained for further statistical analysis [36].

### 2.7. Statistical Analysis

Statistical analyses were performed using Statistical Program for Social Sciences (SPSS 25.0 for Windows, SPSS, IBM Corporation, Armonk, NY, USA) and MetaboAnalyst 5.0 software. Continuous variables were compared using the Mann–Whitney U test. Categorical variables were compared using the Pearson correlation coefficient or Fisher’s exact test. A *p* value < 0.05 was considered statistically significant. A *p* value filter was used to identify differences in normalised intensity between NASH with mild liver fibrosis and NASH with significant liver fibrosis. LC–MS/MS data were adjusted for fold change cutoff points of 1, and the *p* value cutoff point was 0.05. To establish a predictive model, we used partial least squares discriminant analysis (PLS-DA) to classify non/mild fibrosis (F0–1) and significant fibrosis (F2–4). Furthermore, a correlation matrix was plotted using the Pearson correlation coefficients to determine the markers in a high-yield predictive model, and a univariate logistic regression was conducted to estimate the risk associated with NASH significant fibrosis. The area under the receiver operating characteristic (AUROC) for each lipid metabolite was obtained, if it was identified as a significant variable in the predictive model. The *p* value of the odds ratio forest plot was <0.05, and the results were presented on a plot of test sensitivity and 1 − specificity.

## 3. Results

### 3.1. Characteristics of Patients in NASH with Mild Liver Fibrosis and NASH with Significant Liver Fibrosis Groups

Among the 60 patients included in the analysis, the mean age was 35.9 years, mean BMI was 40.8 kg/m^2^, 34 (56.7%) were female, 14 (23.3%) had diabetes mellitus, 19 (31.7%) had hypertension, 30 (50%) had F0–1 fibrosis, and 30 (50%) had F2–F4 fibrosis. As shown in Table 1, patients in the F2–4 group exhibited a higher BMI, fasting glucose level, C-peptide level, AST level, ALT level, GGT level, APRI, FIB-4 score, LSM, and US fatty and fibrosis score. In addition, this group had a higher proportion of males and patients with diabetes than did the F0–F1 group.

### 3.2. Lipidomic Profile Differentiation between NASH Stages

**Figure 1:**
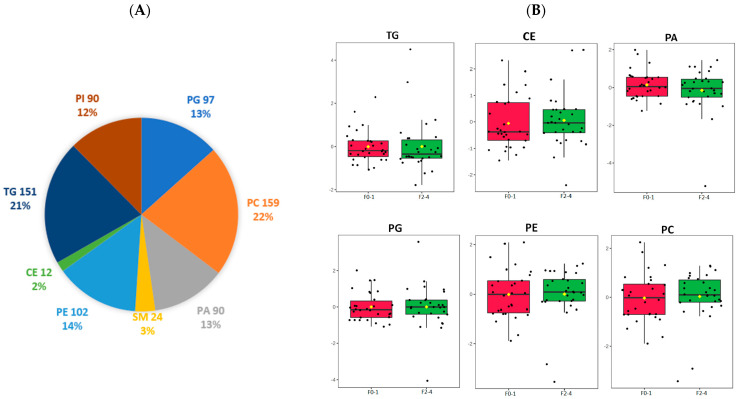
Lipidomic identification of liver lipid subclass (**A**) Distribution of significant lipid subclasses TG, SM, PG, PA, PE, PC, PI, CE; (**B**) Normalized relative abundance presented as median ± quartile of selected lipid species grouped by F0–1, F2–4. TG: triglyceride; SM: sphingomyelin; PG: phosphatidylglycerol; PA: phosphatidic acid; CE: cholesterol ester; PE: phosphatidylethanolamine; PC: phosphatidylcholine; PI: phosphatidylinositol; F0–1, grade 0–1 fibrosis; F2–4, grade 2–4 fibrosis.

We analysed 60 liver samples and, in total, 1379 lipid metabolites were detected, identified, and quantified including 725 species of TG, cholesterol ester (CE), PA, phosphatidylcholine (PC), phosphatidylethanolamines (PE), phosphatidylinositol (PI), phosphatidylglycerol (PG), and sphingomyelin (SM). PC had the highest number of species identified (*n* = 159), followed by TG (*n* = 151) and PE (*n* = 102). The distribution of the lipid classes is depicted in Figure 1A. The normalised relative abundances of individual lipid classes were analysed in the F0–F1 and F2–F4 groups (Figure 1B). No significant difference was observed.

**Figure 2:**
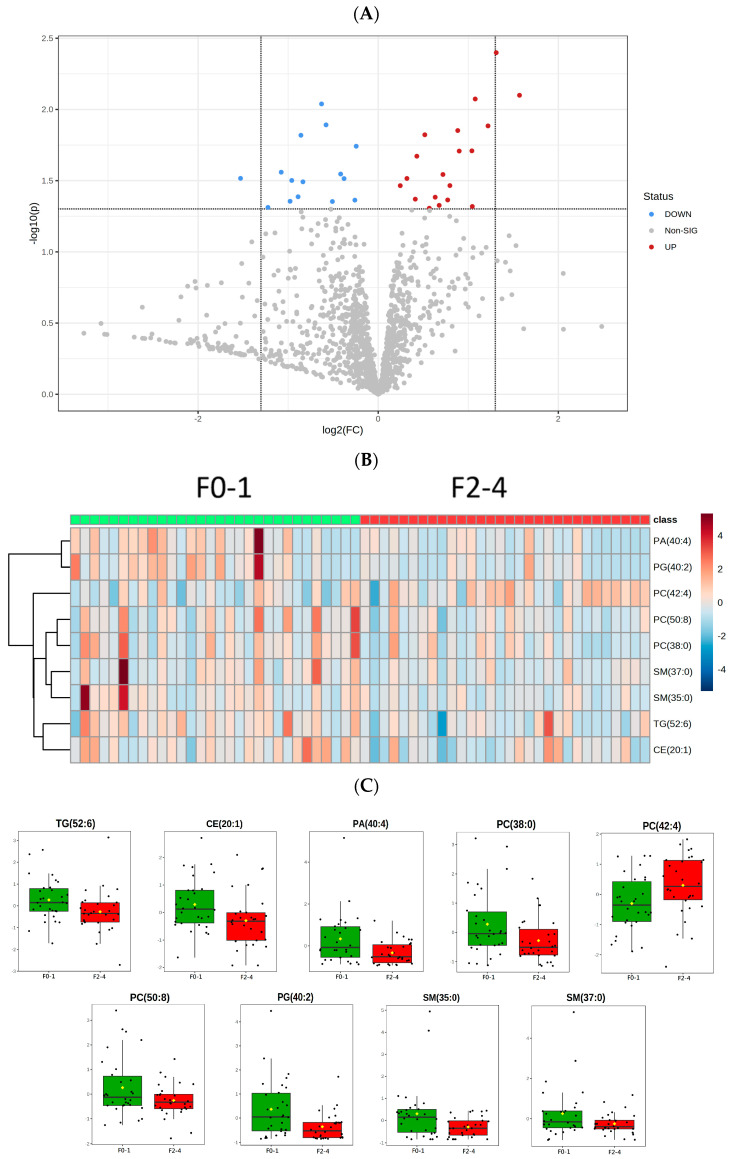
Differentiation of the significant lipid species in two groups. (**A**) The volcano plot illustrates the fold change in x axis and *p* value < 0.05 in y axis. The red dots indicate the fold change over 1 between NASH F0–1 and F2–4, while the blue dots represent the negative fold change.; (**B**) Heat maps of the F0–1/F2–4 for significant change lipids, with rows describing lipid species and columns representing NASH fibrosis samples.; (**C**) Normalized relative abundance presented as median ± quartile of the 9 significant lipid species (*p* < 0.05) in NASH F0–1 vs. F2–4.

Lipid alteration between the two groups is presented on a volcano plot (Figure 2A) with a *p* value < 0.05 and fold change <1. Of the metabolites, CE (20:1); TG (52:6); PC (38:0), (42:4), and (50:8); SM (35:0) and (37:0); PA (40:4); and PG (40:2) had significantly lower concentrations in patients with NASH with stage 2–4 fibrosis than in those with stage 0–1 fibrosis. The heatmap illustrates these significant lipid differences in patients with NASH with fibrosis and depicts the differences in abundance between the fibrosis groups (Figure 2B. However, the concentration of PC (42:4) was higher in the F2–F4 group (Table 2, Figure 2C).

### 3.3. Association of Lipid Metabolites and NAFLD Parameters among Patients with NASH

**Figure 3:**
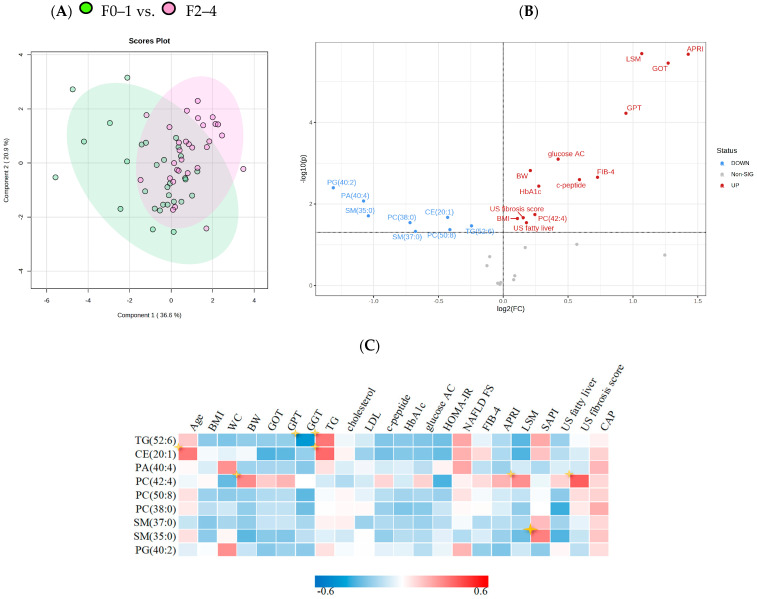
PLS–DA analysis and correlations between significant lipid species and clinical characteristics (**A**) PLS-DA plot analysis of liver lipidomic differentiating patients with F0–1/F2–4. (**B**) Volcano plot of lipid metabolites and clinical characteristics of NASH. X axis shows fold changes for NASH F2–4/F0–1, y axis represents *p* value cut off point (0.05). (**C**) Correlation matrix calculated using Pearson correlation, indicating 13 significant lipid metabolites and key baseline characteristics. Colour corresponds to coefficient value between −0.6 and 0.6.

The distribution of total lipids across the two groups is presented in a PLS-DA plot and stratified by grade of fibrosis (Figure 3A). In the plot, samples are scattered across different areas, indicating different datasets. We used Pearson correlation to calculate the association between selected significant lipid species and biochemical parameters. The correlations of the lipid species with clinical, metabolic features and with NAFLD parameters are presented in Figure 3B,C; Figure 3B shows a volcano plot for fold changes of 1 and *p* values < 0.05. Of the chosen lipidomes, TG (52:6) and CE (20:1) are positively correlated with serum triglyceride level, NAFLD-FS, and splenic arterial pulsatility index (SAPI). SAPI is a diagnostic and predictive index for chronic liver fibrosis obtained by doppler sonography, suggesting a correlation with hepatic fibrosis. The heatmap also shows that older patients are more likely to have liver CE (20:1) accumulation. Unlike the other lipid species, PC (42:4) had a positive correlation with body weight and LSM and especially US fibrosis score, indicating its potential as a biomarker for severe fibrosis.

### 3.4. Predictive Models of NASH Fibrosis in Terms of Levels of Lipid Metabolites

**Figure 4:**
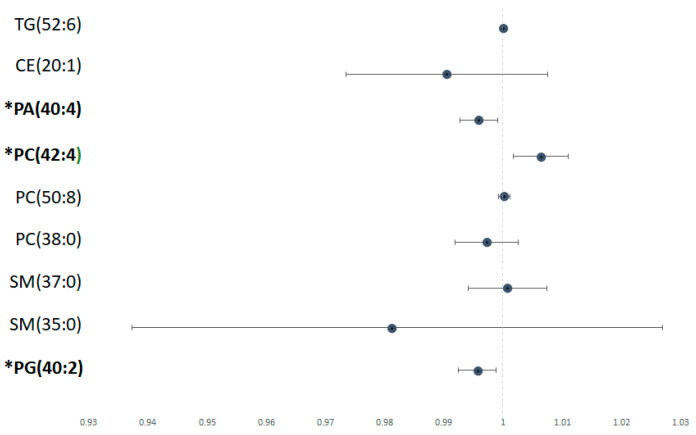
Risk of lipid metabolite with significant versus non/mild fibrosis Odds ratio of each significant lipid species calculated using univariate logistic regression analysis, depicted in forest plot. Metabolites labelled with * have a statistically significant *p* value (*p* < 0.05).

Using logistic regression univariate analysis, we found that several lipid species were associated with significant liver fibrosis (Figure 4). In the liver, lower PG (40:2) and PA (40:4) were associated with higher risk of severe liver fibrosis (*p* < 0.05). Conversely, higher levels of PC (42:4) were associated with higher risk of significant liver fibrosis.

**Figure 5:**
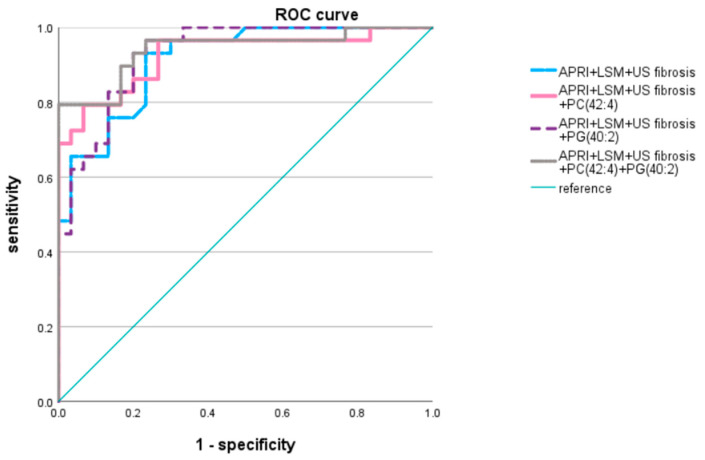
Area under receiver operating curve of models Lipid metabolites with adjusted *p* value < 0.05 were applied to statistical model. APRI, LSM, US fibrosis score, PC (42:4) and PG (40:2) were applied to predictive model.

Multiple predictive models were developed based on a correlation matrix and dimorphic data for NASH fibrosis detection. We selected the liver fibrosis-related indicators including non-invasive serum markers and APRI and image diagnosing techniques, such as LSM and US fibrosis score. The AUROC was 0.914 (*p* < 0.0001; Table 3, Figure 5). Among the 13 selected lipid metabolites, PG (40:2), PA (40:4), and PC (42:4) were associated with a decreased NASH fibrosis risk as mentioned above; thus, we constructed predictive models with one among various permutations of these lipid subclasses. The results revealed that PA (40:4) and PC (42:4) in combination with APRI, LSM, and US fibrosis score had higher area under the curve (AUC) values (AUROC = 0.925, 0.93; *p* < 0.0001) than the other lipid metabolites. The predictive model with APRI, LSM, PC (42:4), and PG (40:2) yielded the highest AUROC (0.941, *p* < 0.0001), with a sensitivity and specificity of 79.3% and 100%, respectively, and a higher positive predictive value (82.8%) and negative predictive value (83.3%) compared to the other three models constructed (Table 4). Because they were part of the best predictive model for fibrosis grade, PC (42:4) and PG (40:2) have potential in the diagnosis and prediction of a patient’s significant fibrosis stage.

## 4. Discussion

This study characterized various lipid spectra accumulated in the livers of patients with NASH with different grades of fibrosis, and we established a predictive model of severity with a high AUC (0.941), sensitivity (79.3%), and specificity (100%). Patients with histopathologically diagnosed NASH with different degrees of fibrosis had different concentrations of liver lipid metabolites, including TG, CE, PC, PE, PG, PI, PA, and SM, which indicates that lipid metabolic status may differ between fibrosis stages, as suggested by previous studies [20].

Triglyceride plays an essential role in liver intracellular lipid accumulation and may be involved in the progression of NAFLD and NASH. The decrease or increase in specific TG species in the liver and plasma might be associated with NAFLD and NASH status [37,38], and substantial change in the presence of steatosis [39]. This study focused on the fibrosis stage of NASH, and identified one TG species, TG (43:0) (unpublished data) and (52:6), whose concentration substantially decreases in severe fibrosis and is positively associated with serum TG level. Although there was a relative difference between the two groups in our study, it did not reach statistical significance. H. Alamri et al. reported an increase of TGs (52:1), (52:2), and (52:3) in patients with NAFLD [40]. Although the results are not consistent with our work, long chain species of TGs may still play an important role in liver metabolism. In a mouse model and patients with NAFLD, TG has been reported to be associated with a protective mechanism to alleviate fibrosis in individuals with obesity and insulin resistance [41,42]. This might suggest that patients with severe fibrosis lack the specific TG species that assist in the metabolism of lipotoxicity.

Although TGs appear to be the most common lipids in the liver, other lipids, such as cholesterol and SM, may contribute to cellular dysfunction [41,43]. Significant elevation of hepatic CE concentrations has been observed in patients with NASH [20]. However, our study showed a clear decrease in CE (20:1) levels in the NASH F2–4 group, which is consistent with the finding of a previous study in which specific CE signatures were lower in cases of NASH versus cases of steatosis [39]. A cohort study found that hepatic free cholesterol increased progressively from patients with normal histology to those with NASH, but total CE did not change [37]. The variation in outcomes may be explained by the different fibrosis stages and steatosis conditions investigated. We also found that CE levels are positively associated with age, which may influence lipid metabolism and warrants consideration [44].

SP (sphingolipid) plays a critical role in plasma membrane composition, and key SPs such as ceramide and sphingosine can influence immune pathways, including tissue damage, cytokine release, and ultimately, fibrotic progression. Liver fibrosis is the replacement of organ tissue and cells by scar and connective tissue cells, accompanied by healing and inflammatory reactions [45]. Several studies have attributed inflammation and liver fibrosis to elevation in specific sphingolipids. Experiments have found that a high fat and cholesterol diet increases hepatic levels of SM and decreases PC level, and expression of SM synthases has been found to be higher in both mice and patients with NASH. Studies have also demonstrated the considerably elevated SM in patients with NASH compared to those without [20,46]. Similarly, plasma SM (36:0) has been reported to regress in patients that have received obesity surgery [47]. However, we observed lower values for patients with significant fibrosis in comparison with those with mild fibrosis, particularly of SM (35:0) and SM (37:0). The association between SM signatures in the liver and plasma and NASH fibrosis remains disputed, and additional factors, such as the levels of specific enzymes, should be considered to increase prediction accuracy.

PC accounts for most of the lipid species identified in our study. The role of PC has been investigated to better understand NAFLD and NASH progression. Furthermore, previous studies have indicated that significant increase in PC levels in type 2 diabetes mellitus patients [48]. Our study identified three PC species, namely PC (38:0), (42:4), and (50:8), that had significantly lower concentrations in the severe fibrosis group. However, unlike the other PC species, PC (42:4) concentration increased in the F2–4 group. Research has produced similar results regarding PC levels when comparing patients with obesity without NAFLD or NASH and those with obesity with NAFLD or NASH [49]. Puri et al. reported that total PC concentrations are lower in patients with NAFLD and patients with NASH than in healthy individuals [37]. Conversely, PC (22:0/18:1) and (26:1/11:0) levels were reported to be higher in the NASH group than in the NAFLD group, whereas PC (22:6/0:0) and (16:1/0:0) levels were lower, suggesting complicated lipid metabolic alteration [21]. Studies have reported that the molar ratio of PC to PE is closely associated with liver disease progression, and that a considerable decrease in liver PC/PE indicates the progression of NAFLD [50,51]. This ratio acts as a better predictor than PC levels alone. PC and PE are the most abundant phosphoglycerates, and future studies can analyse these components individually or in combination for more accurate results.

Numerous biomarkers that predict liver fibrosis in patients with NAFLD/NASH have been investigated in various studies, including those using image techniques, such as LSM, SAPI, and CAP; noninvasive serum markers, such as the APRI, FIB-4 score, and FLI; and other metabolism-related data. The combined use of APRI and FibroScan-based scores yield accurate predictions of NASH/NAFLD liver fibrosis [16]. Other metabolism-based markers have also exhibited more than 80% specificity and sensitivity in the prediction of significant fibrosis [52,53]. This study found an AUROC of 0.914 for APRI, LSM, and US fibrosis scores, which were positively correlated with liver fibrosis, and the sensitivity and specificity were 93.1% and 76.7%, respectively, for F2–4 fibrosis. PC (42:4) and PG (40:2) also had a high AUROC value of 0.941 in combination with fibrosis-related indices, indicating their high predictive accuracy.

This study has some limitations. First, variations in biopsy sampling may have been present, and the sampling area may have affected lipid identification. The findings are the average results of various liver cell type, and periportal and pericentral hepatocytes could be distinguished with different metabolic activities. Second, despite several findings of lipidomic species demonstrating statistically significant differences, concentrations of odd-chain fatty acids were low and nearly undetectable in human tissue; thus, such results were not further discussed. Third, problems remain in using only liver lipidomic profiles as biomarkers for fibrosis diagnosis, and detailed identification of the mechanisms behind lipid changes requires further investigation. Studies have indicated that NASH is strongly associated to specific genetic change, such as *PNPLA3*, yet the factor was not classified in our work [54]. A larger study group with long-term follow-up to determine prognosis remains necessary. However, our observations contribute to further research in the mechanisms of NASH fibrosis and assist in determining optimal prediction methods for preventive intervention.

## 5. Conclusions

This study demonstrated that the changes in the concentrations of particular lipid species of TG, CE, SM, PC, PG, PA, PI and PE in the liver are correlated with NASH fibrosis stage, and the levels of these species may indicate hepatic steatosis regression or progression in patients with morbid obesity. The establishment of a predictive model facilitates the study of NASH progression and prognosis prediction, with substantial predictive values suggesting high accuracy in practical use if these are confirmed in further lipidomic investigations.

## Figures and Tables

**Table 1:** Demographic and biochemical data between NASH with F0–F1 and NASH with F2–F4 patients.

	All (*n* = 60)	NASH with F0–F1 (*n* = 30)	NASH with F2–F4 (*n* = 30)	*p* Value
Age, years *	35.9 ± 7.7	37.2 ± 8.5	34.6 ± 6.8	0.168
Sex (M/F) (%)	26/34 (43.3/56.7)	8/22 (26.7/73.3)	18/12 (60.0/40.0)	0.009
Smoking (yes/no) (%)	9/51 (15.0/85.0)	7/23 (23.3/76.7)	2/28 (6.7/93.3)	0.590
BMI, kg/m^2^ *	40.8 ± 5.3	39.2 ± 4.7	42.3 ± 5.5	0.011
WC, cm *	120.9 ± 11.8	118.7 ± 11.4	123.5 ± 11.8	0.166
HTN (yes/no) (%)	19/41 (31.7/68.3)	8/22 (26.7/73.3)	11/19 (36.7/63.3)	0.405
HTN under medication (yes/no)	10/9 (52.6/47.4)	5/3 (62.5/37.5)	4/7 (57.1/42.9)	-
DM (yes/no) (%)	14/46 (23.3/76.7)	2/28 (6.7/93.3)	12/18 (40.0/60.0)	0.002
DM under medication (yes/no)	7/7 (50/50)	1/1 (50/50)	6/6 (50/50)	-
Biochemical data				
Fasting glucose, mg/Dl *	118.7 ± 38.7	102.9 ± 18.5	134.0 ± 46.7	0.009
HOMA-IR *	6.1 ± 11.4	3.2 ± 3.3	9.99 ± 16.4	0.046
C-peptide, ng/mL	4.6 ± 2.1	3.8 ± 1.2	5.3 ± 2.4	0.025
Cholesterol, mg/dL *	195.6 ± 41.7	200.3 ± 49.4	191.1 ± 32.8	0.097
LDL, mg/dL *	134.3 ± 34.0	135.0 ± 41.1	133.6 ± 26.5	0.729
TG, mg/dL *	187.4 ± 152.2	192.1 ± 110.3	182.8 ± 185.9	0.255
Total bilirubin, mg/dL *	0.7 ± 0.3	0.6 ± 0.2	0.8 ± 0.8	0.101
AST, U/L *	44.2 ± 33.0	25.9 ± 9.7	62.5 ± 37.9	<0.001
ALT, U/L *	66.1 ± 42.7	45.1 ± 21.4	90.0 ± 48.4	<0.001
GGT, U/L *	50.6 ± 23.1	41.4 ± 22.3	59.3 ± 2075	0.008
Creatinine, mg/dL *	0.7 ± 0.2	0.7 ± 0.1	0.7 ± 0.2	0.273
Albumin, g/dL *	4.5 ± 0.3	4.5 ± 0.3	4.4 ± 0.3	0.351
Platelet, 1000/mm^3^ *	279.8 ± 64.0	297.0 ± 59.5	262.6 ± 64.8	0.054
Non-invasive serum markers				
APRI *	0.42 ± 0.34	0.23 ± 0.11	0.62 ± 0.39	<0.001
NAFLD-FS *	2.44 ± 1.31	2.25 ± 1.37	2.64 ± 1.23	0.190
FIB-4 score *	0.72 ± 0.43	0.52 ± 0.22	0.95 ± 0.49	<0.001
FLI *	84.13 ± 31.38	89.96 ± 10.36	78.3 ± 38.6	0.242
Imaging techniques				
LSM (E score) *, kPa	9.7 ± 6.4	6.9 ± 3.3	12.5 ± 7.5	<0.001
CAP, dB/m	323.2 ± 66.6	320.2 ± 36.2	326.4 ± 89.0	0.111
US fatty score *	7.3 ± 1.6	6.8 ± 1.7	7.7 ± 1.4	0.046
US fibrosis score *	5.0 ± 0.7	5.0 ± 0.7	5.6 ± 1.1	0.035
SAPI	0.80 ± 0.24	0.80 ± 0.22	0.90 ± 0.26	0.082

NASH, non-alcoholic steatohepatitis; LSM, liver stiffness measurement; CAP, controlled attenuation parameter; HTN, hypertension; DM, diabetes mellitus; BMI, body mass index; SD, standard deviation; M, male; F, female; WC, waist circumference; LDL, low-density lipoprotein; TG, triglyceride; AST, aspartate aminotransferase; ALT, alanine aminotransferase; GGT, gamma-glutamyltransferase; HOMA-IR, the homeostasis model assessment of insulin resistance; APRI, aspartate aminotransferase/platelet ratio index; FLI, fatty liver index; NAFLD-FS, nonalcoholic fatty liver disease fibrosis score; FIB-4 score, Fibrosis-4 score; US, ultrasonographic; SAPI, splenic arterial pulsatility index. * Expressed as mean ± standard deviation.

**Table 2:** Demographic data between NASH with F0–F1 and NASH with F2–F4 patients.

Lipid Class	Lipid Species	*p* Value	Fold Change
Triglyceride (TG)	TG (52:6)	0.034281	1.185
Cholesterol ester (CE)	CE (20:1)	0.021292	1.3462
Phospholipid (PL)	PA (40:4)	0.00844	2.1097
	PC (38:0)	0.028609	1.6457
	PC (42:4)	0.018124	0.8443
	PC (50:8)	0.043317	1.3303
	PG (40:2)	0.003993	2.4802
Sphingolipid (SP)	SM (35:0)	0.019529	2.0565
	SM (37:0)	0.047131	1.5988

NASH, non-alcoholic steatohepatitis; TG, triglyceride; SP, sphingolipid; PL, phospholipid; TG, triglyceride; CE, cholesterol ester; PA, phosphatidic acid; PC, phosphatidylcholine; PE, phosphatidylethanolamines; PI, phosphatidylinositol; PG, phosphatidylglycerol; SM, sphingomyelin.

**Table 3:** AUROC curves and statistical classification models for NASH F2–4.

Model	C Statistic 95%CI	*p* Value
APRI + LSM + US fibrosis score	0.914 (0.846–0.982)	<0.0001
APRI + LSM + US fibrosis score + PC (42:4)	0.925 (0.856–0.995)	<0.0001
APRI + LSM + US fibrosis score + PG (40:2)	0.930 (0.869–0.991)	<0.0001
APRI + LSM + US fibrosis score + PC (42:4) + PG (40:2)	0.941 (0.88–1.003)	<0.0001

Lipid metabolites with an adjusted *p* value < 0.05 were applied to the statistical model. APRI, aspartate aminotransferase/platelet ratio index; LSM, liver stiffness measurement; US, ultrasonographic.

**Table 4:** Diagnostic accuracies of models for NASH F2–4.

Model	Sensitivity (%)	Specificity (%)	PPV (%)	NPV (%)
APRI + LSM + US fibrosis score	93.1	76.7	79.3	76.7
APRI + LSM + US fibrosis score + PC (42:4)	79.3	93.3	86.2	80
APRI + LSM + US fibrosis score + PG (40:2)	86.2	87.7	89.7	80
APRI + LSM + US fibrosis score + PC (42:4) + PG (40:2)	79.3	100	82.8	83.3

PPV, positive predictive value; NPV, negative predictive value; APRI, aspartate aminotransferase/platelet ratio index; LSM, liver stiffness measurement US, ultrasonographic.

## Data Availability

Not applicable.
